# Supplementary material for: Genetic polymorphisms lead to major, locus-specific, variation in piRNA production in mouse
Source: EMBO J. 2025 Jun 5;44(14):4104–19. doi: 10.1038/s44318-025-00475-4 (PMC12264111; doi:10.1038/s44318-025-00475-4)
Supplement: Supplementary file 1 — Appendix [file 44318_2025_475_MOESM1_ESM.pdf]

Appendix for

Genetic polymorphisms lead to major, locus-specific, variation in piRNA production in mouse.

Eduard Casas[1,2,4*], Adrià Mitjavila--Ventura[1,2,3*], Pío Sierra[5*], Cristina Moreta-Moraleda[6,7], Judith Cebria[8], Ilaria Panzeri[9], J. Andrew Pospisilik[9], Josep C. Jimenez-Chillaron[7,8,10], Sonia V. Forcales [6,11$], Tanya Vavouri[1,2,5$]

1 Josep Carreras Leukaemia Research Institute (IJC), Ctra de Can Ruti, Camí de les Escoles s/n, 08916, Badalona, Barcelona, Spain.
2 Germans Trias i Pujol Research Institute (IGTP), Can Ruti Campus, Badalona, Barcelona 08916, Spain.
3 Autonomous University of Barcelona (UAB), Barcelona, Spain.
4 University of Barcelona (UB), Barcelona, Spain.
5 Open University of Catalunya (UOC), Spain.6 Department of Pathology and Experimental Therapeutics, School of Medicine and Health Sciences, Campus of Bellvitge, University of Barcelona, Carrer de la Feixa Llarga, s/n, L'Hospitalet de Llobregat, Barcelona, Spain.
7 Oncobell Program, Bellvitge Biomedical Research Institute (Idibell), Gran Via de les Corts Catalanes L'Hospitalet de Llobregat, Barcelona, Spain.
8 Institut de Recerca Sant Joan de Déu, Endocrine Division, Esplugues de Llobregat, Barcelona, Spain. 08950.
9 Center for Epigenetics, Van Andel Research Institute, Grand Rapids, MI 49503, USA.
10 Department of Physiological Sciences, School of Medicine, University of Barcelona, Carrer de la Feixa Llarga, s/n, L'Hospitalet de Llobregat, Barcelona, Spain.
11 Serra Húnter Professor, Department of Pathology and Experimental Therapeutics, School of Medicine and Health Sciences, Campus of Bellvitge, University of Barcelona, Carrer de la Feixa Llarga, s/n, L'Hospitalet de Llobregat, Barcelona, Spain.
*Contributed equally.
[$]Corresponding authors.

Table of contents

1. **Appendix Figure S1** **Page 2**
2. **Appendix Figure S2** **Page 3**
3. **Appendix Figure S3** **Page 4**
4. **Appendix Figure S4** **Page 6**
5. **Appendix Figure S5** **Page 10**
6. **Appendix Figure S6** **Page 11**
7. **Appendix Figure S7** **Page 12**
8. **Appendix Figure S8** **Page 14**
9. **Appendix Figure S9** **Page 15**
10. **Appendix Figure S10** **Page 16**
11. **Appendix Figure S11** **Page 17**
12. **Appendix Figure S12** **Page 18**

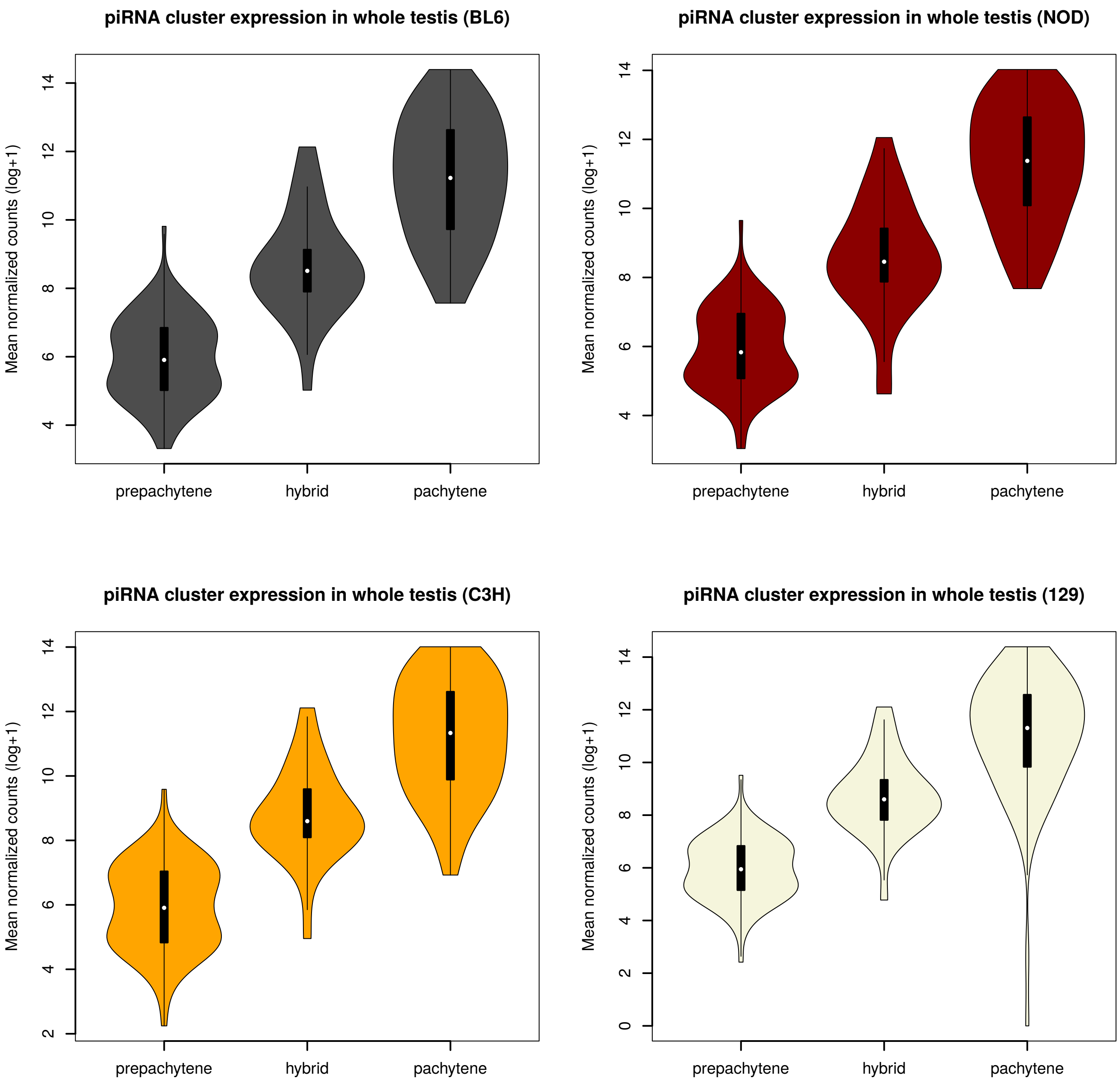

Appendix Figure S1. Abundance of piRNAs from prepachytene, pachytene and hybrid piRNA cluster in whole testis samples of the four inbred mouse strains. The classification of the three sets of clusters were retrieved from (Ding et al, 2018) .

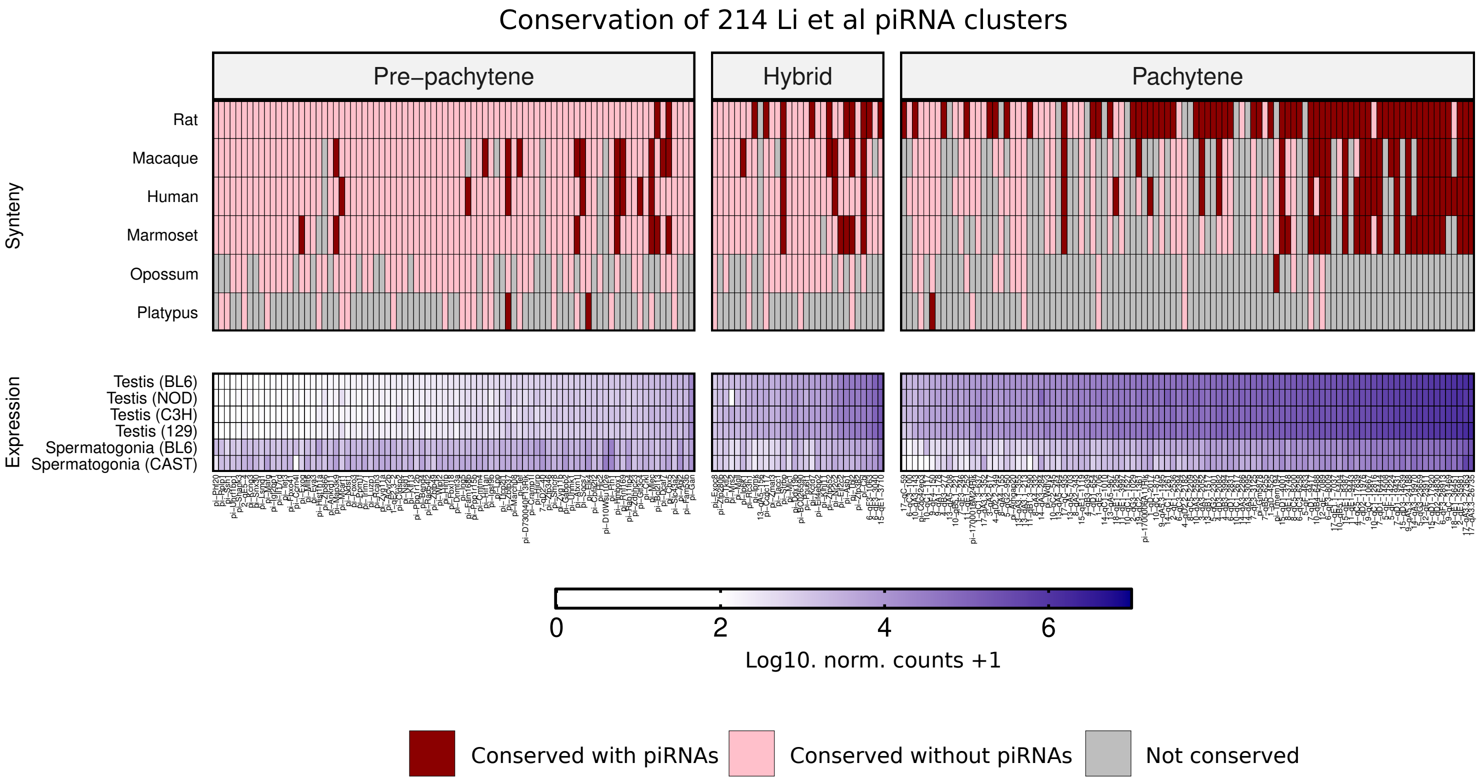

Appendix Figure S2. Annotation of conservation and expression of 214 piRNA clusters in other mammals and in testis and spermatogonia samples of five inbred strains. Conservation and expression data for other mammals retrieved from (Özata et al, 2020).

A

Pairwise correlation of sRNA expression across testis samples
piRNA clusters annotated by Li et al. (2013)

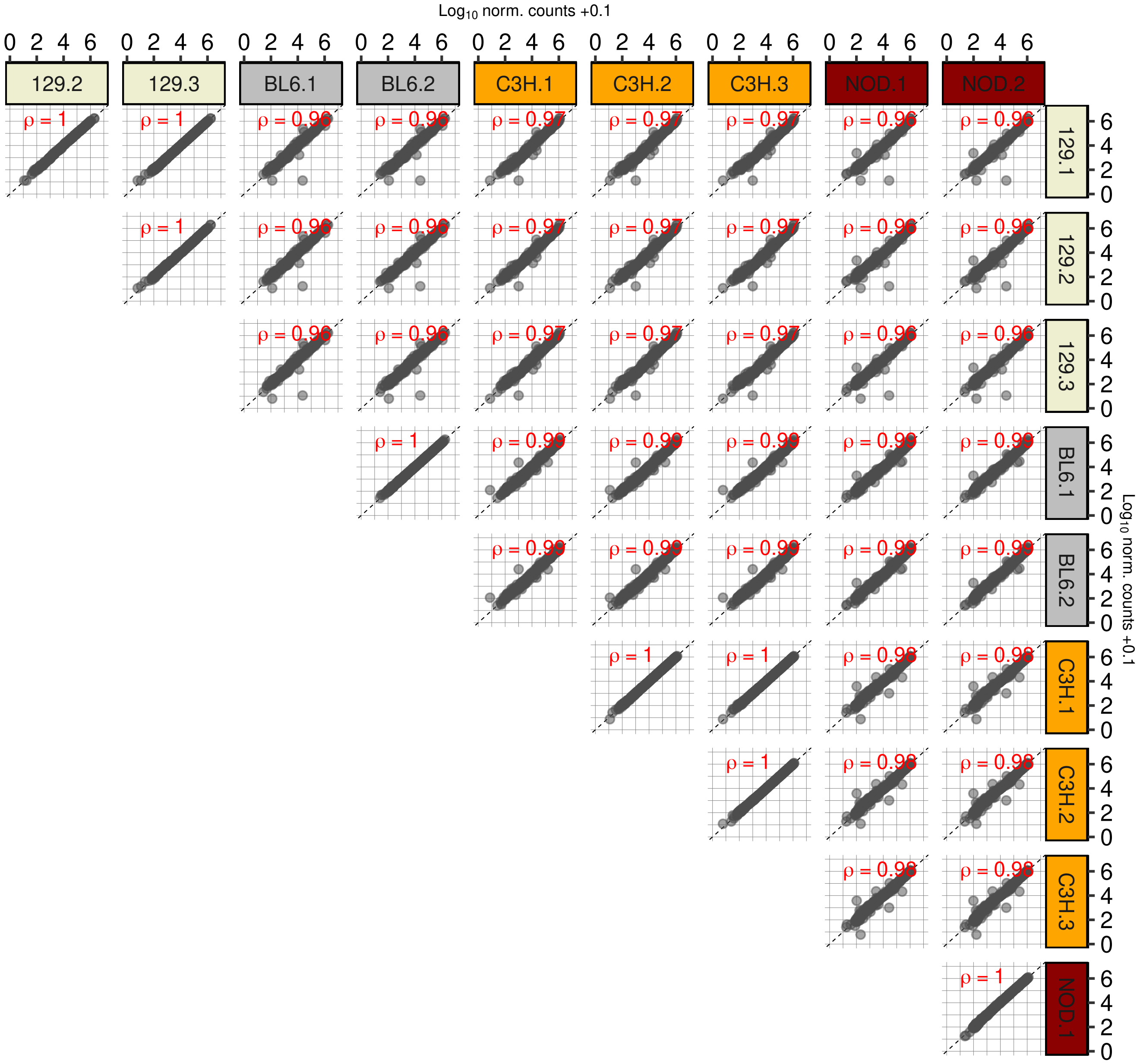

B

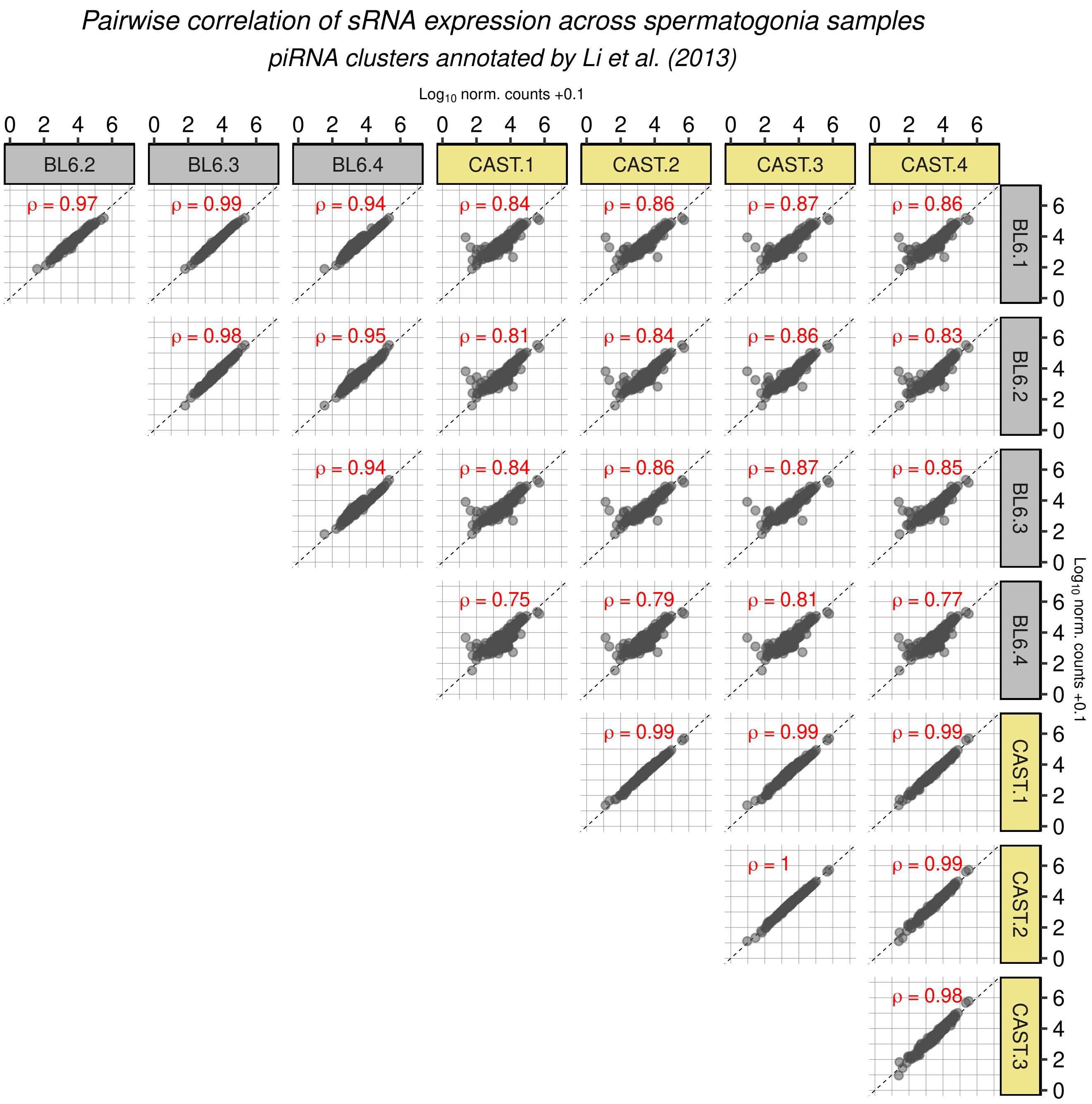

Appendix Figure S3. Correlation of piRNA cluster expression between all mouse samples from inbred mouse strains.
(A) Pairwise correlations of piRNA expression between testis samples
(B) Pairwise correlations of piRNA expression between spermatogonia samples.

A

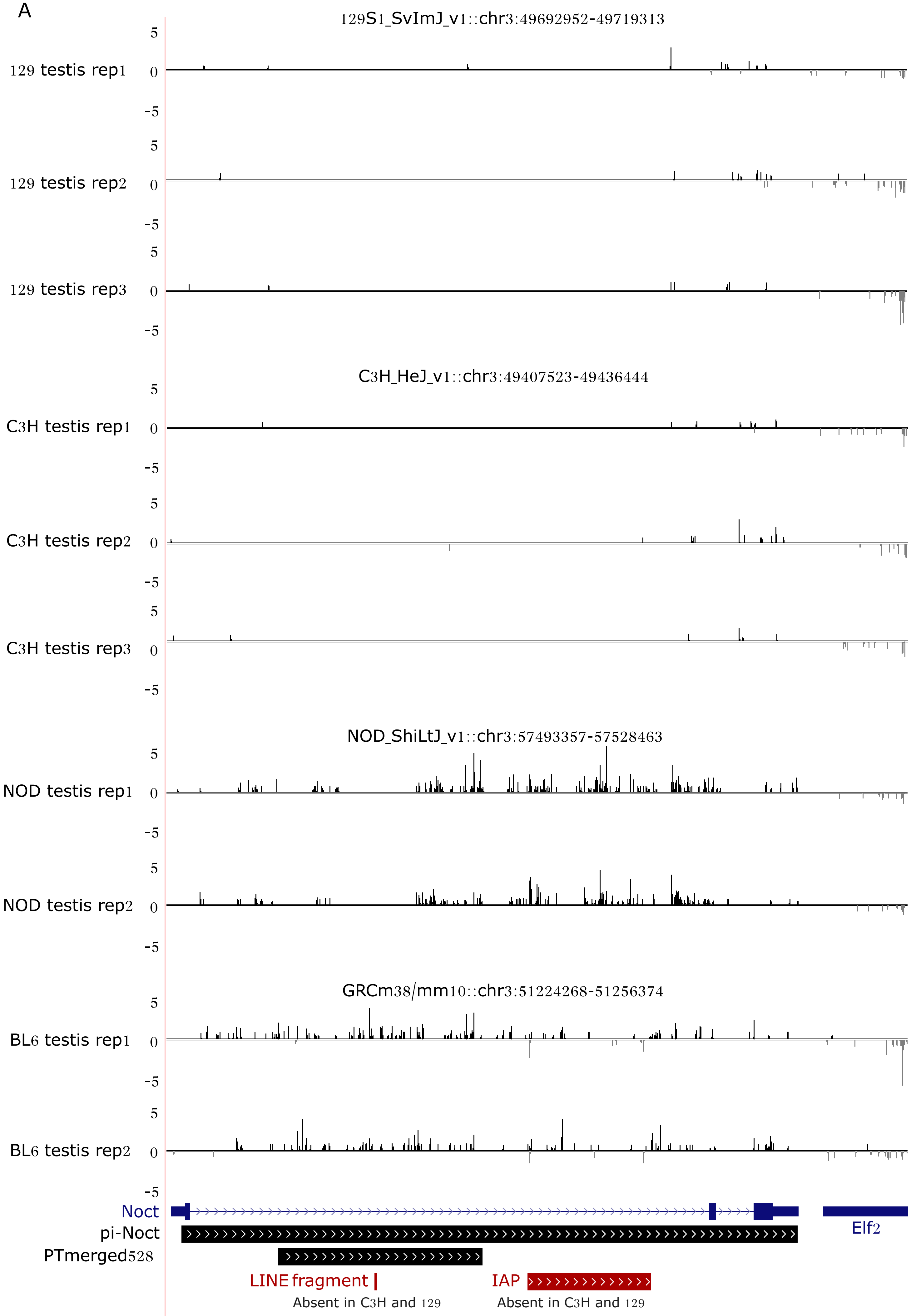

129S1_SvImJ_v1::chr3:49692952-49719313
129 testis rep1
129 testis rep2
129 testis rep3
C3H_HeJ_v1::chr3:49407523-49436444
C3H testis rep1
C3H testis rep2
C3H testis rep3
NOD_ShiLtJ_v1::chr3:57493357-57528463
NOD testis rep1
NOD testis rep2
GRCm38/mm10::chr3:51224268-51256374
BL6 testis rep1
BL6 testis rep2
5
0
-5
Noct
pi-Noct
PTmerged528
Elf2
LINE fragment
Absent in C3H and 129
IAP
Absent in C3H and 129

B

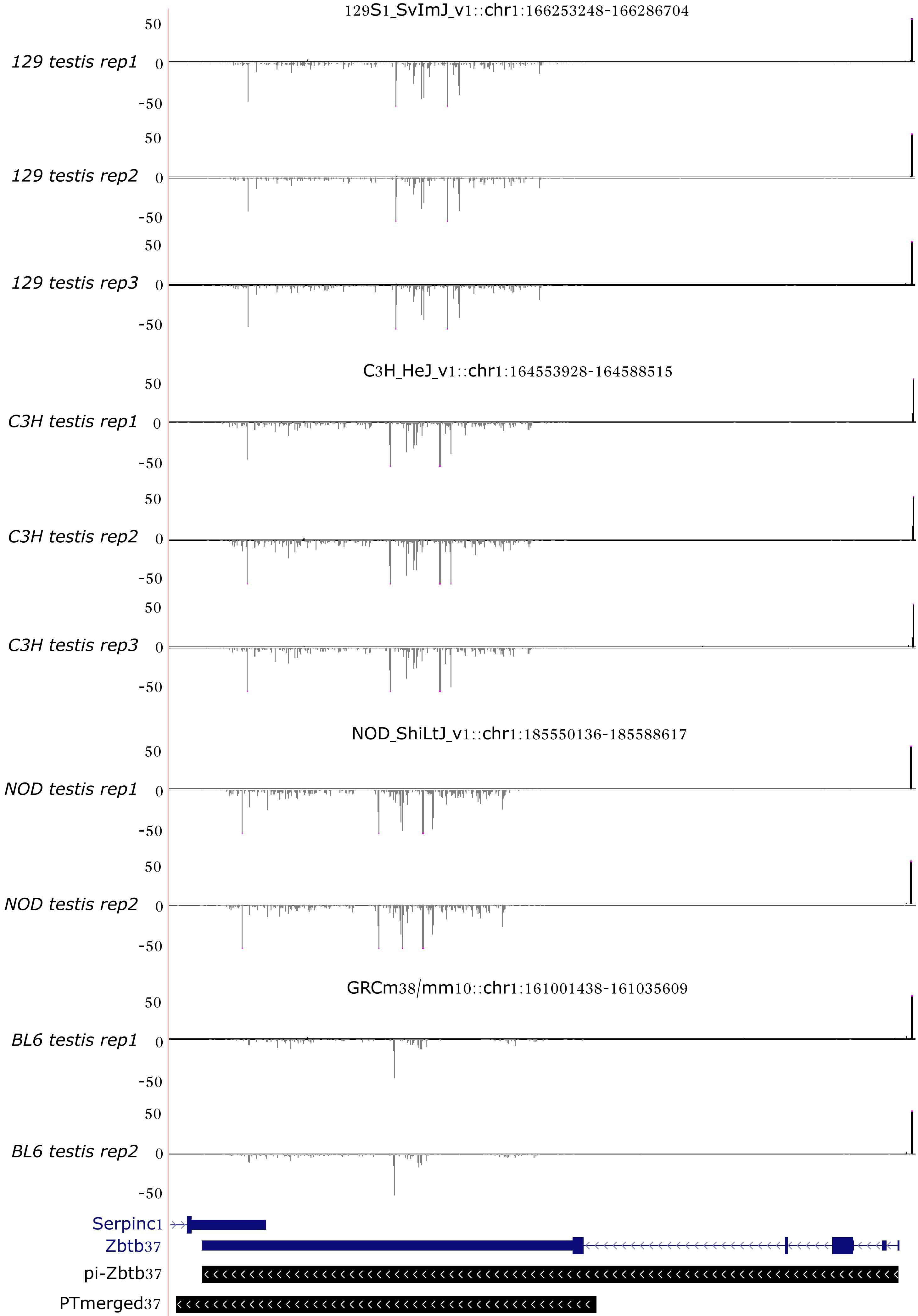
129S1_SvImJ_v1::chr1:166253248-166286704
129 testis rep1
129 testis rep2
129 testis rep3
C3H_HeJ_v1::chr1:164553928-164588515
C3H testis rep1
C3H testis rep2
C3H testis rep3
NOD_ShiLtJ_v1::chr1:185550136-185588617
NOD testis rep1
NOD testis rep2
GRCm38/mm10::chr1:161001438-161035609
BL6 testis rep1
BL6 testis rep2
50
0
-50
Serpinc1
Zbtb37
pi-Zbtb37
PTmerged37

C

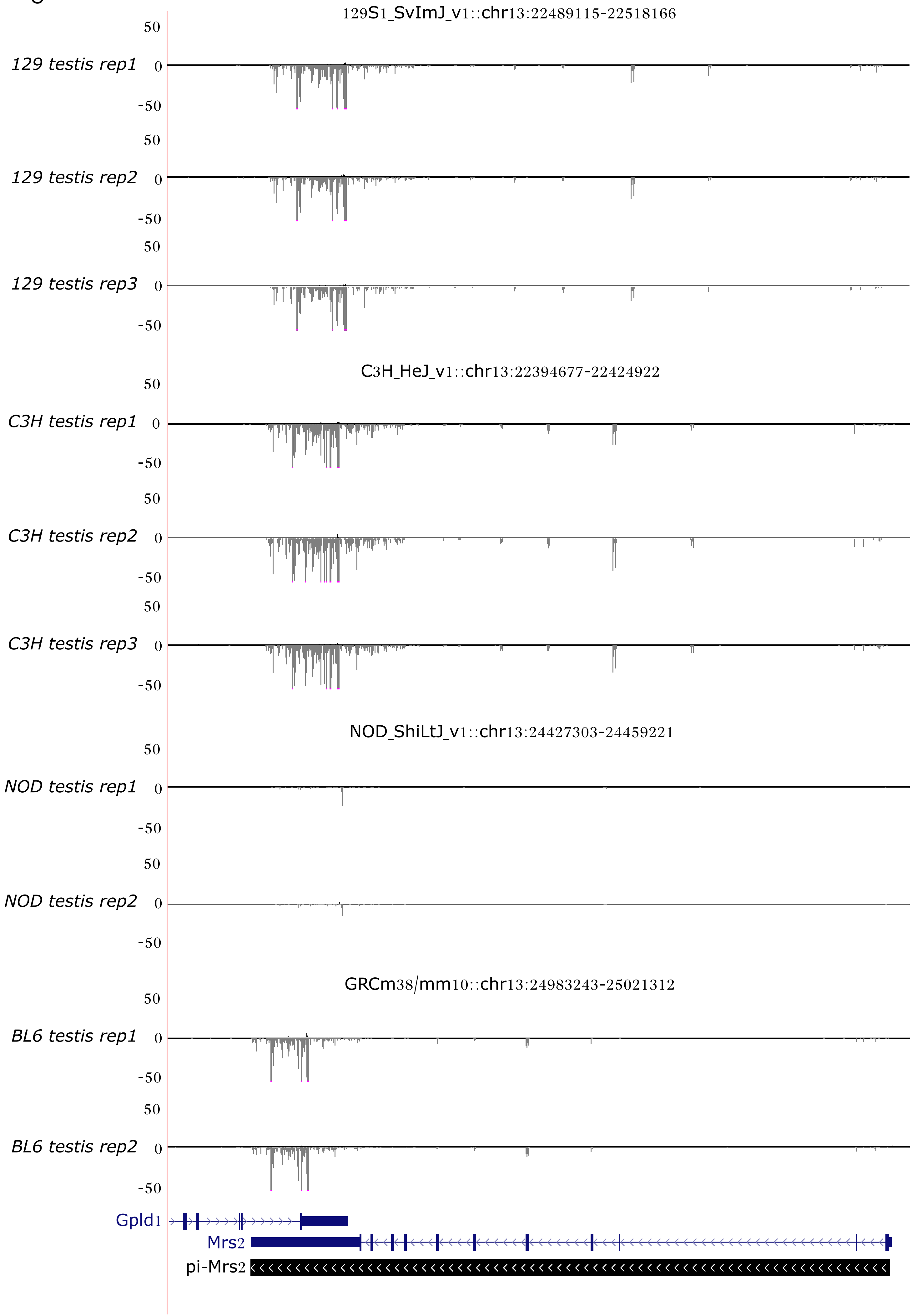

129S1_SvImJ_v1::chr13:22489115-22518166
129 testis rep1
129 testis rep2
129 testis rep3
C3H_HeJ_v1::chr13:22394677-22424922
C3H testis rep1
C3H testis rep2
C3H testis rep3
NOD_ShiLtJ_v1::chr13:24427303-24459221
NOD testis rep1
NOD testis rep2
GRCm38/mm10::chr13:24983243-25021312
BL6 testis rep1
BL6 testis rep2
50
0
-50
Gpld1
Mrs2
pi-Mrs2

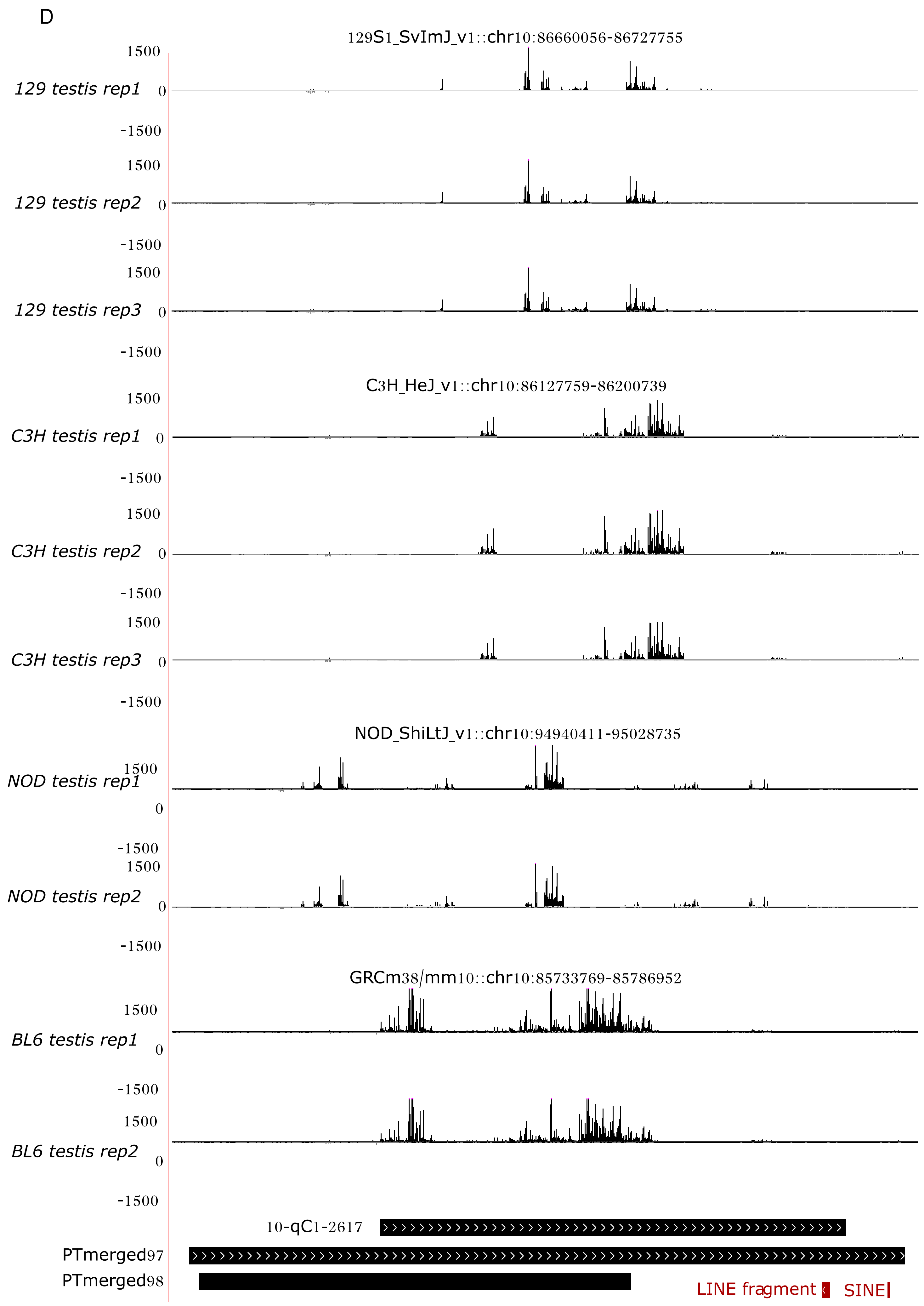

Appendix Figure S4. Classical inbred mouse strains produce significantly different levels of piRNAs from (A) pi- Noct (also known as pi-Ccrn4l), (B) pi-Zbtb37, (C) pi-Mrs2 and (D) 10-qC1-2617.

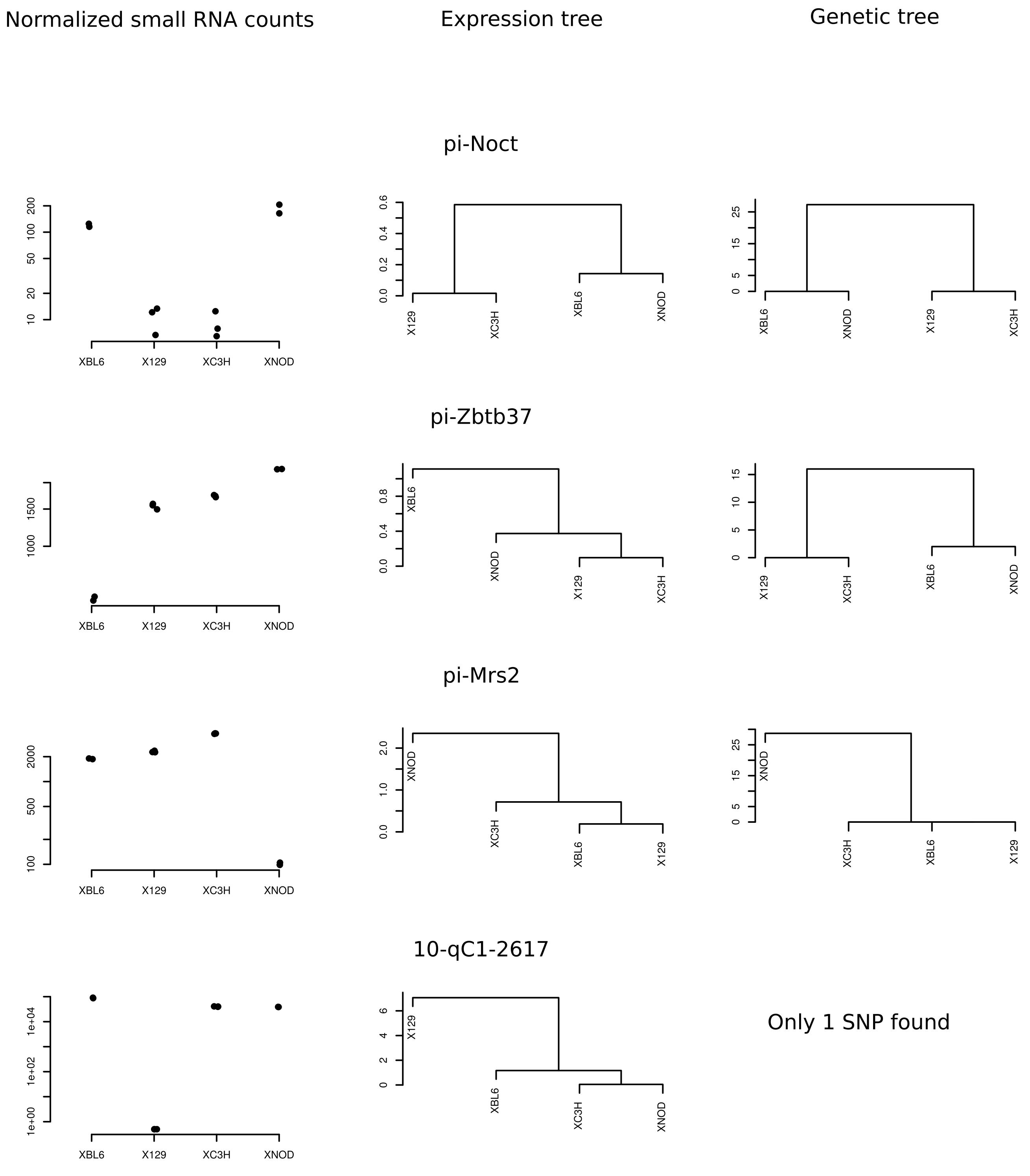

Appendix Figure S5. Comparison of genetic and expression distance tree for piRNA clusters shown in Fig 1E.

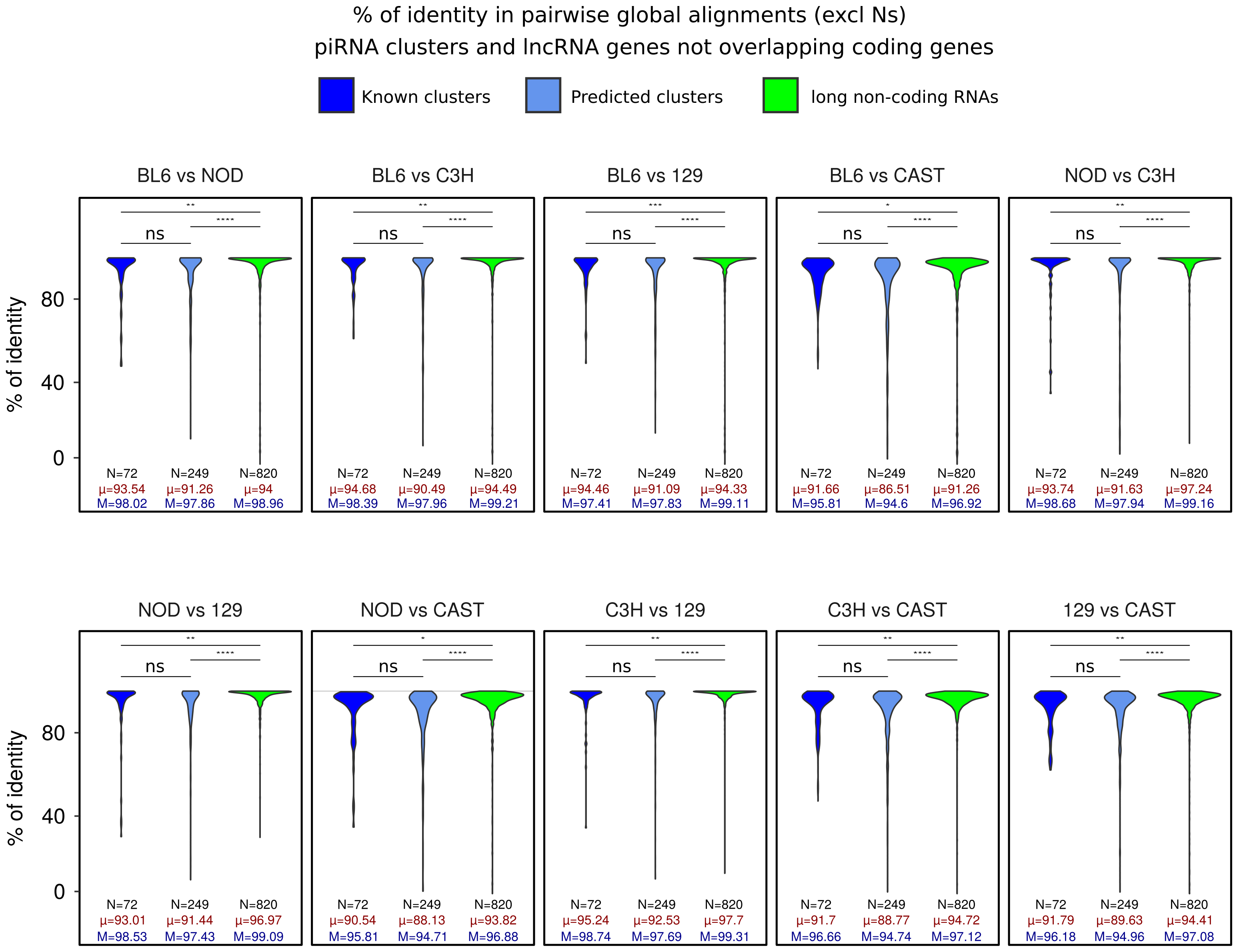

Appendix Figure S6. Comparison of inter-strain sequence identity between known, noncoding piRNA-producing loci, predicted non-coding piRNA-producing loci and long non-coding RNAs. The significance of the differences in the distributions was calculated using the Wilcoxon rank-sum test (ns not significant, * <0.05, ** < 0.01, *** <0.001).

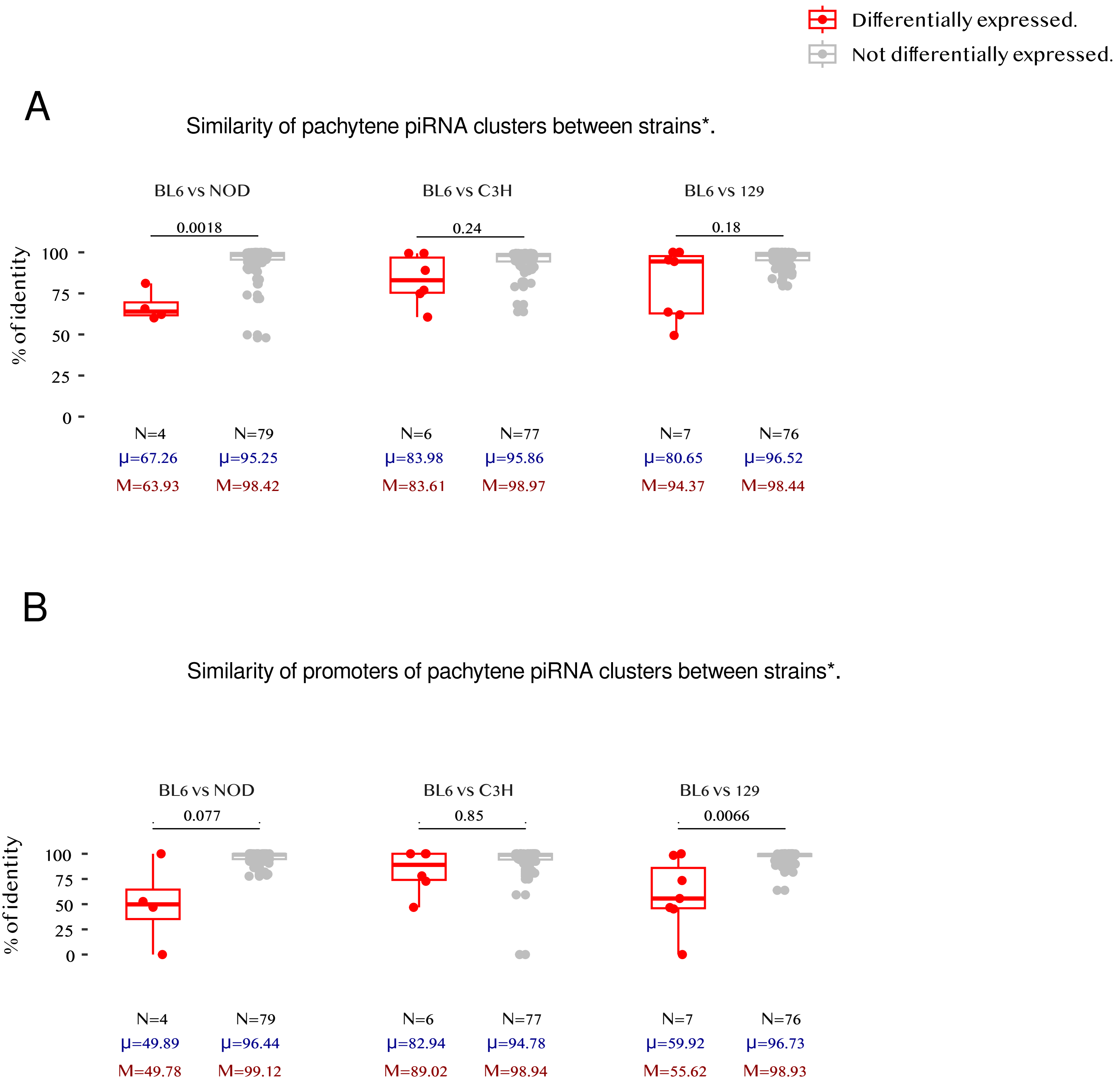
Differentially expressed.
Not differentially expressed.
A
Similarity of pachytene piRNA clusters between strains*.
BL6 vs NOD
BL6 vs C3H
BL6 vs 129
0.0018
0.24
0.18
% of identity
100
75
50
25
0
N=4
μ=67.26
M=63.93
N=79
μ=95.25
M=98.42
N=6
μ=83.98
M=83.61
N=77
μ=95.86
M=98.97
N=7
μ=80.65
M=94.37
N=76
μ=96.52
M=98.44
B
Similarity of promoters of pachytene piRNA clusters between strains*.
BL6 vs NOD
BL6 vs C3H
BL6 vs 129
0.077
0.85
0.0066
% of identity
100
75
50
25
0
N=4
μ=49.89
M=49.78
N=79
μ=96.44
M=99.12
N=6
μ=82.94
M=89.02
N=77
μ=94.78
M=98.94
N=7
μ=59.92
M=55.62
N=76
μ=96.73
M=98.93

C

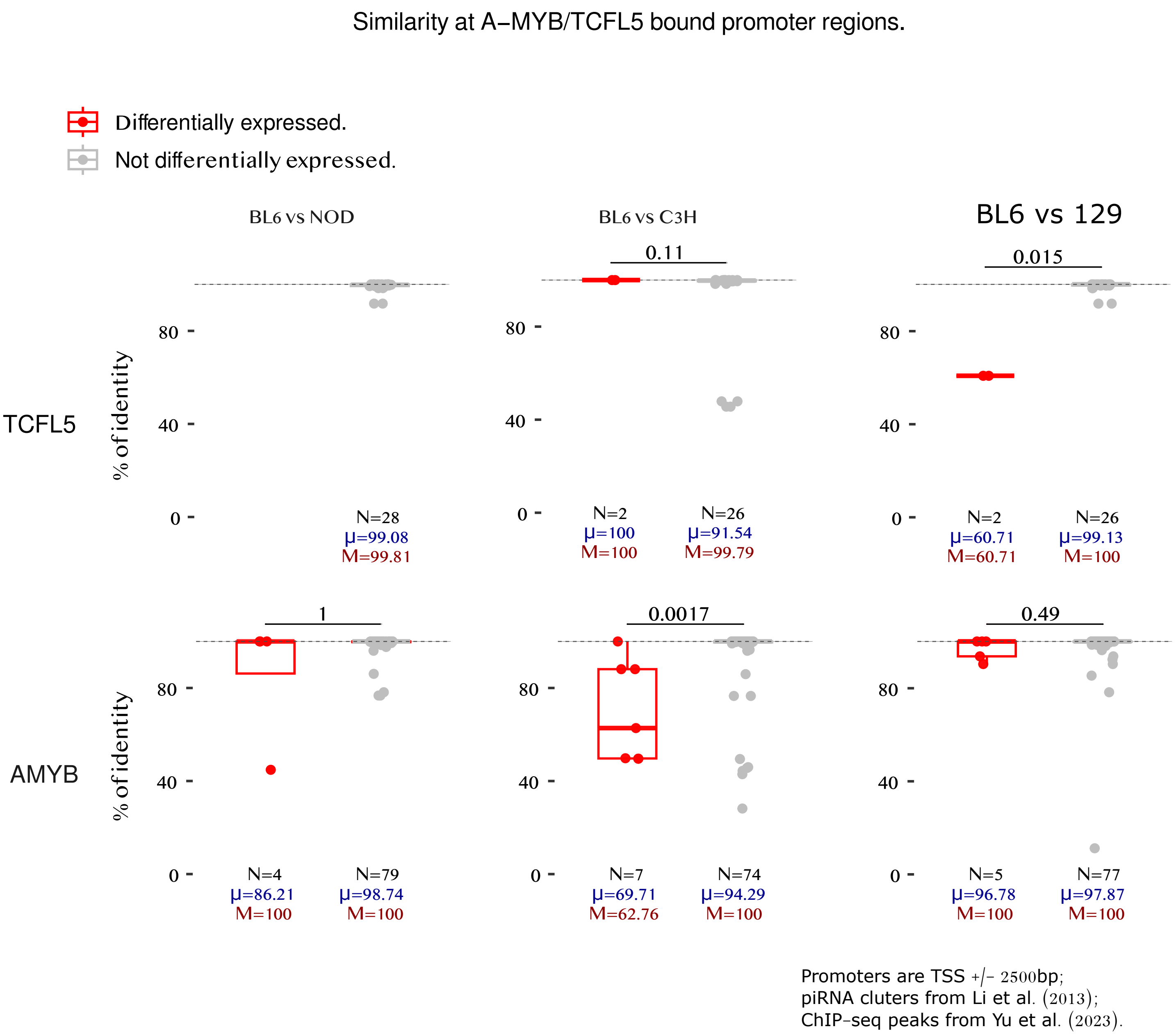

Appendix Figure S7. Inter-strain sequence identity among differentially expressed and not differentially expressed piRNA clusters along the full locus (A), at promoters (B) and at AMYB and TCFL5 transcription factor binding sites (C).

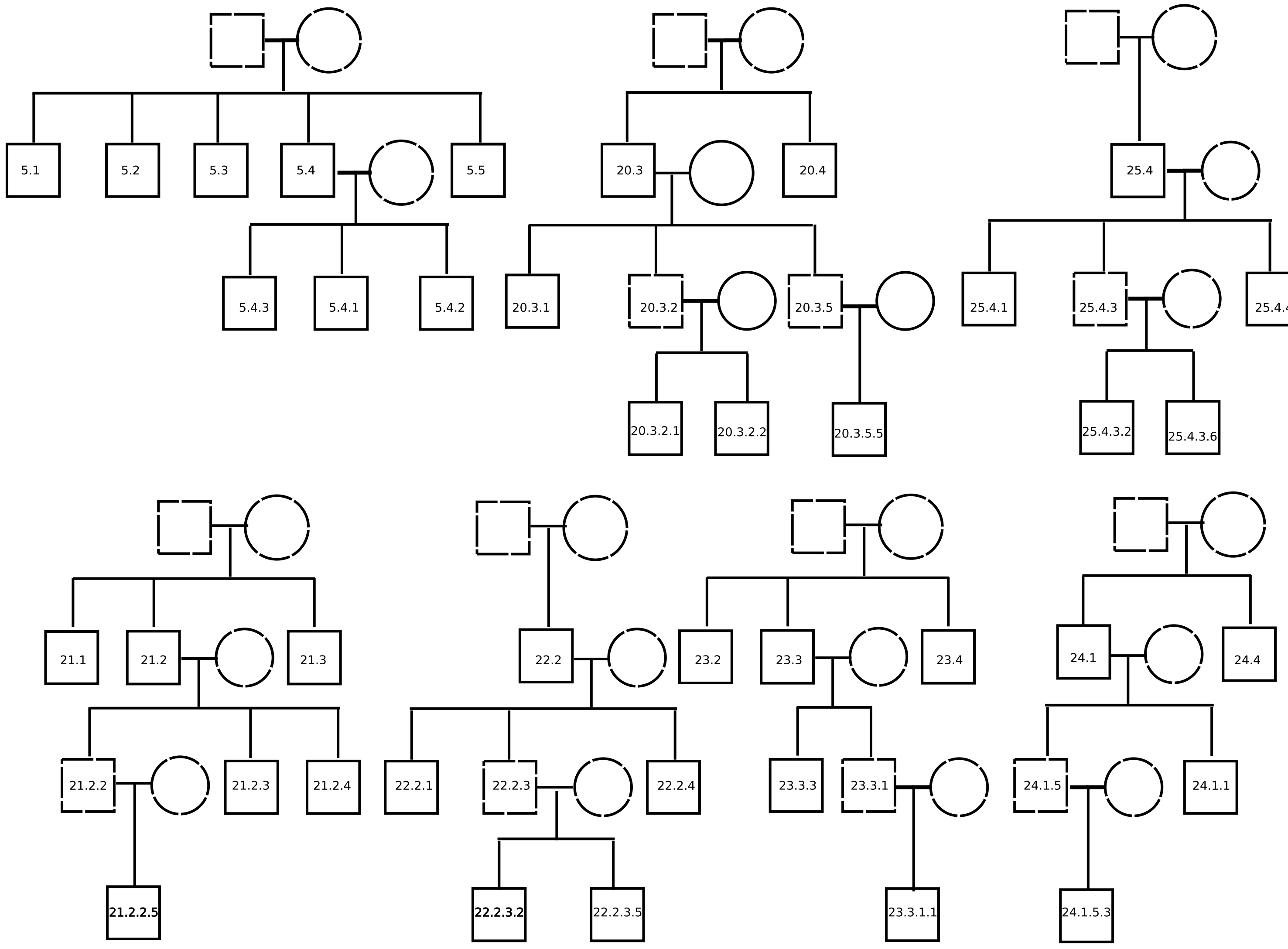

Appendix Figure S8. Pedigrees showing the relatedness of mice of the ICR strain from which small RNAs were sequenced and analysed. Dotted lines indicate animals from which we do not have data. The identifiers of the samples are shown inside the figure and correspond to samples indicated in Table EV1.

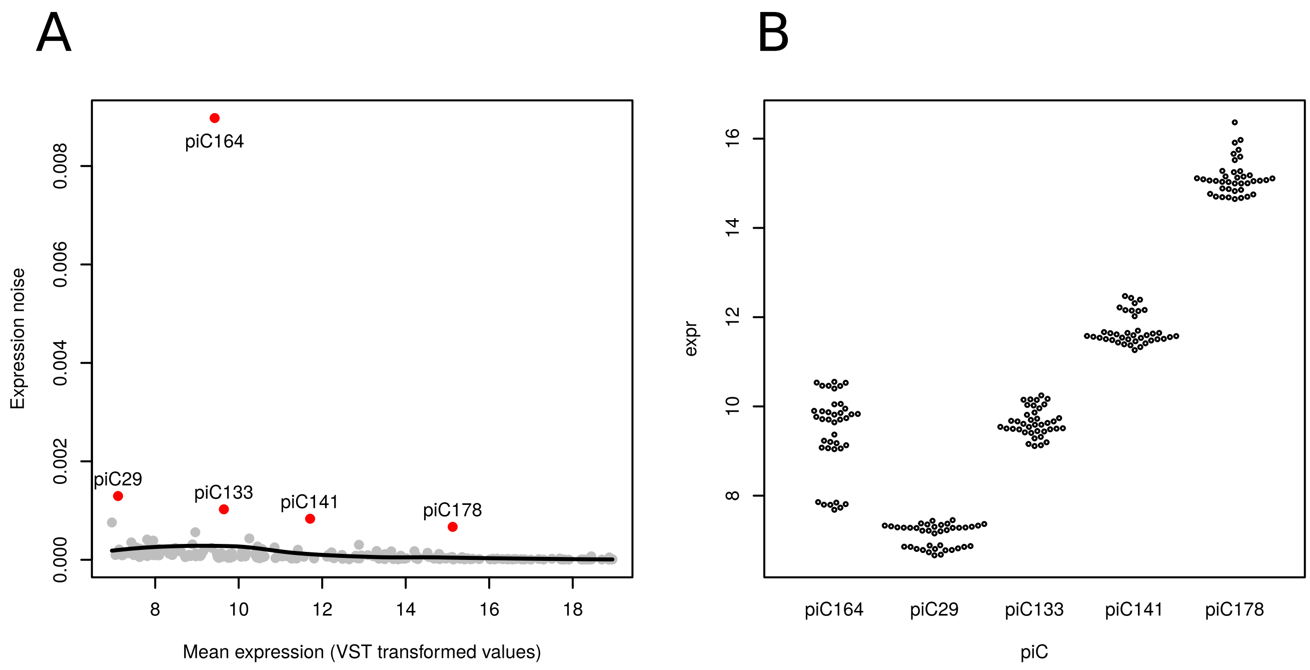

Appendix Figure S9. (A) piRNA clusters with higher than expected “noise” in expression among ICR mice were identified by plotting the coefficient of variation squared against mean expression and then identifying the genes with the largest residuals. The top five most variable piRNA clusters are shown in red. The piRNA cluster expression values were adjusted for differences in library size, transformed using the VST transformation and corrected for batch. (B) Transformed expression values for the top 5 most variable piRNA clusters in the 39 ICR mice.

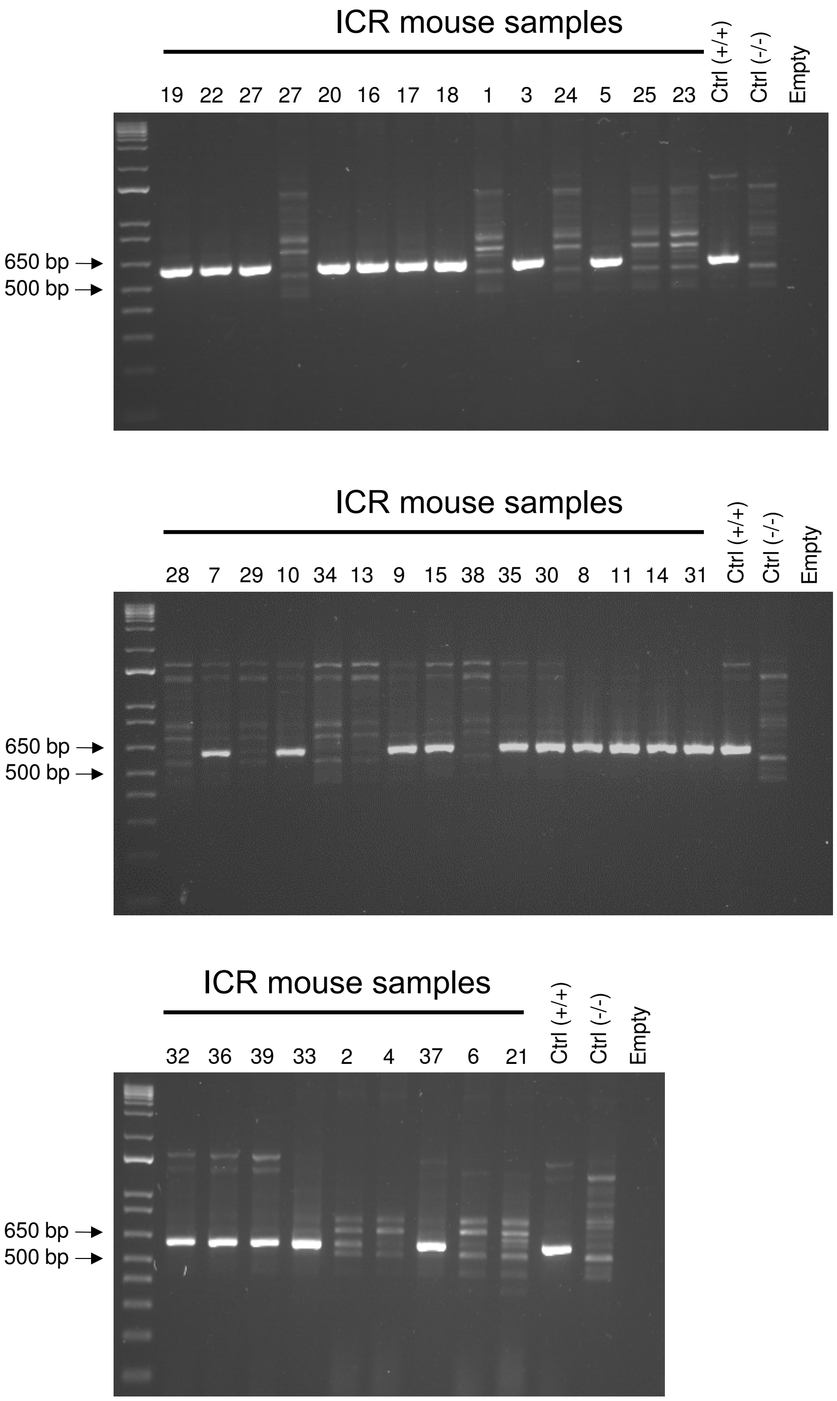

Appendix Figure S10. Genotyping of Noct IAP in mice. T The samples used for the genotyping were from the same mice as those used to generate small RNA sequencing data (labelled sample01-sample06 and sample16- sample27 in Table EV1). The forward primer is located inside the IAP element whereas the Reverse primer is located outisde the IAP. Noct IAP-containing genomes generate an amplicon of 610bp.

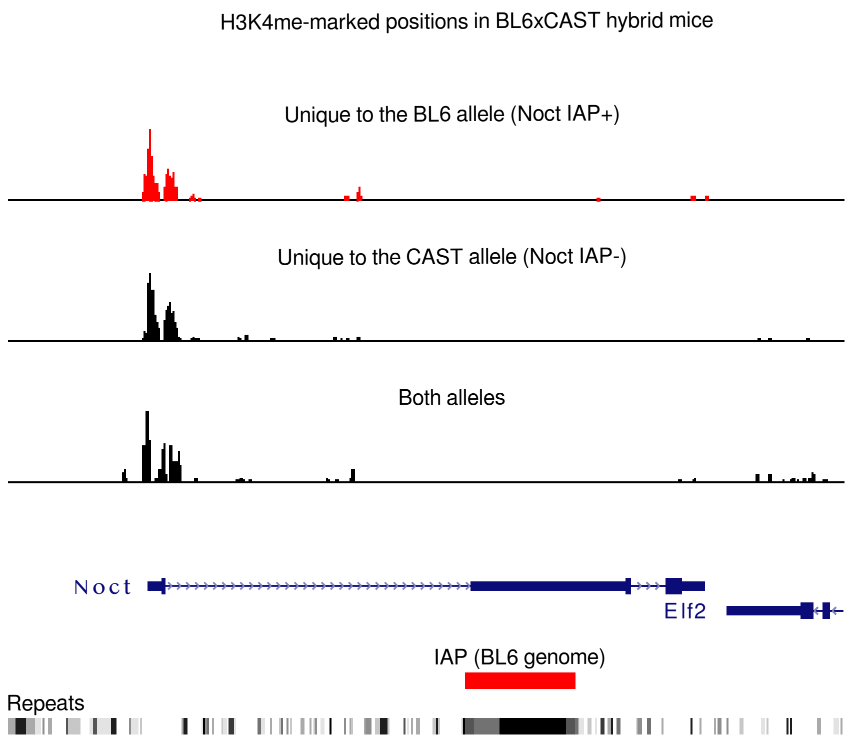

Appendix Figure 11. Allele-specific analysis of H3K4me3 ChIP-Seq from BL6xCAST F1 hybrid mice shows that the active H3K4me3 chromatin mark is found at similar levels on both Noct alleles. Reads mapping unambiguously to each of the two alleles using strain-specific single nucleotide polymorphisms (SNPs) are shown on the top two tracks. Uniquely mapping reads that do not overlap strain-specific SNPs are shown in the bottom track.

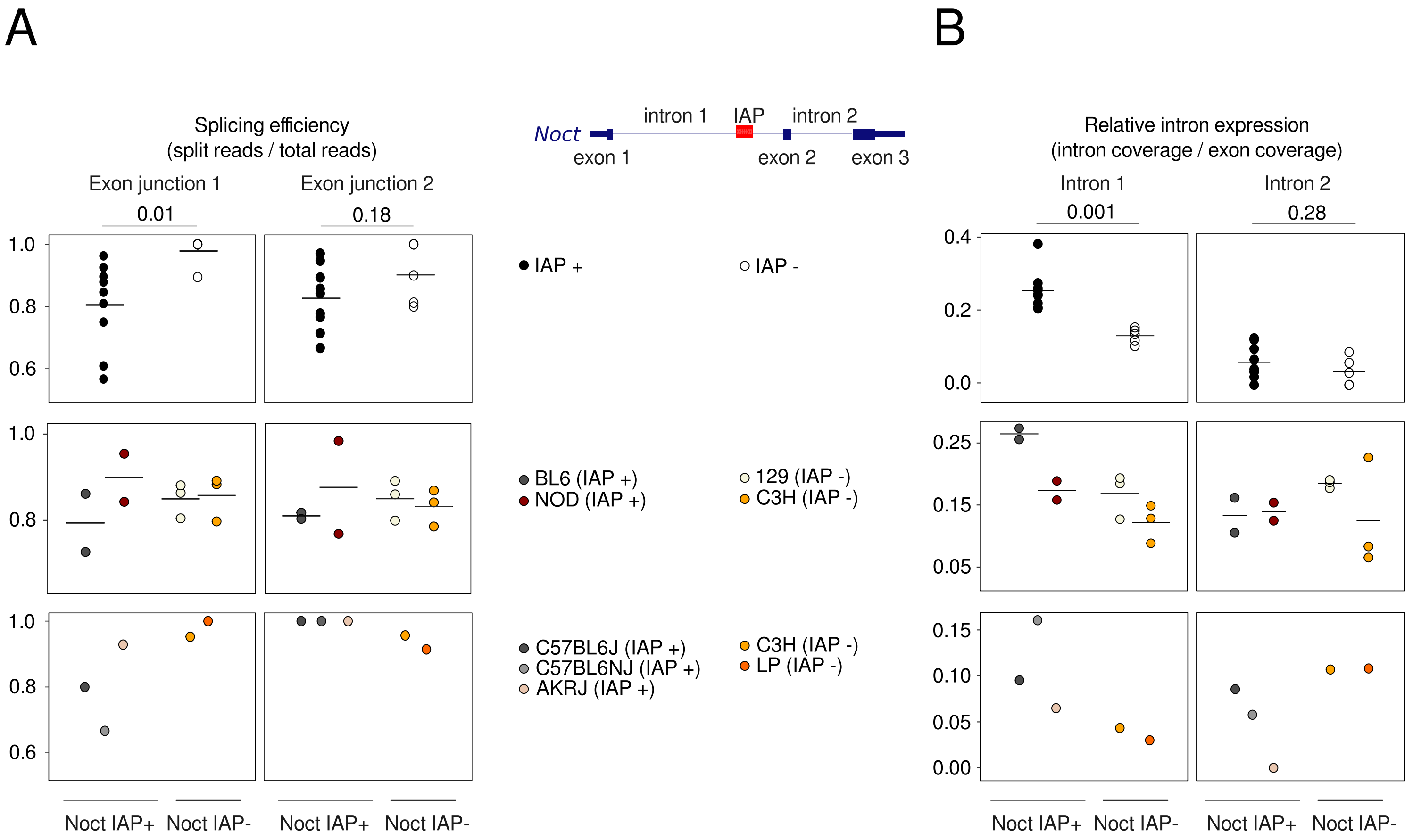

Appendix Figure S12. Comparison of Noct intron retention between genotypes. Comparison of (A) splicing efficiency and (B) relative intron expression between mouse genotypes with/without the IAP in the first intron of Noct. The datasets analysed are testis total RNA-seq data from fourteen genotyped ICR mice (9 Noct IAP+ and 5 Noct IAP-) (top panels), from four inbred strains BL6 (Noct IAP+), NOD (Noct IAP+), 129 (Noct IAP-) and C3H (Noct IAP-) (middle panels) and from C57BL/6J (Noct IAP+ strain), C57BL/6NJ (Noct IAP+ strain), AKR/J (Noct IAP+ strain), C3H/HeJ (Noct IAP- strain) and LP/J (Noct IAP- strain) data from (Yu et al, 2019) (bottom panels). The significance of the differences in the comparisons of distributions of splicing efficiency and intron retention values, between mice with the Noct IAP versus mice without it, is calculated using the Wilcoxon rank sum test and the p-values are indicated.
